# CD4+ T cell recognition of HIV-1 alternate reading frame proteins

**DOI:** 10.3389/fimmu.2025.1600132

**Published:** 2025-05-22

**Authors:** Joel Sop, Tyler P. Beckey, Joel N. Blankson

**Affiliations:** Department of Medicine, Johns Hopkins Medicine, Baltimore, MD, United States

**Keywords:** human immunodeficiency virus (HIV), alternate reading frame, CD4+ T cell, latency, cytokine secretion

## Abstract

HIV-1 alternative reading frame proteins (ARFPs) have been shown to elicit CD8+ T cell responses, but less is known about the recognition of these proteins by CD4+ T cells. In this study, we analyzed responses of CD8-depleted peripheral blood mononuclear cells from chronic progressors (CPs) on suppressive antiretroviral therapy to ARFP peptide pools derived from HIV Gag, polymerase (Pol), and envelope (Env) proteins. Memory CD4+ T cell responses were detected to Gag ARFP peptide pools in 7 out of 13 CPs and to Env ARFP peptide pools in 2 out of 13 CPs. Individual peptide stimulation identified immunogenic peptides that were predicted to bind to major histocompatibility complex class II (MHC-II) proteins. HIV RNA was detected in culture supernatants from 3 of 6 CPs following stimulation of CD4+ T cells with ARFP peptide pools. These findings demonstrate that ARFP-derived peptides elicit antigen-specific CD4+ T cell responses and may contribute to latency reversal. Our data expand the known HIV immunopeptidome and suggest that ARFPs may serve as potential targets for immune-based interventions.

## Introduction

HIV-1 alternative reading frame proteins (ARFPs) have been reported to elicit CD8+ T cell responses (1–3); however, their recognition by CD4+ T cells and their broader role in antiviral immunity remain unclear. ARFPs arise through alternative translation mechanisms, including ribosomal frameshifting and leaky scanning, and have been identified in multiple viral families, including retroviruses, flaviviruses, and coronaviruses (4–8). The identification of sORFs (small open reading frames) in noncanonical translation events has expanded the known repertoire of viral proteins, with implications for antigen presentation and immune recognition (9). In HIV-1, ARFPs have been detected from several genes, yet their biological significance remains unclear. Evidence suggests that ARFPs may influence viral replication, immune evasion, and antigen presentation, raising questions about their immunogenicity and functional relevance (1, 3).

Previous studies have primarily focused on CD8+ T cell responses to ARFPs (1–3), but whether these proteins can be processed and presented via the MHC class II pathway to stimulate CD4+ T cells remains an open question. Given that noncanonical HIV-1 peptides have been found to elicit T cell responses in other viral infections, the potential for ARFPs to serve as CD4+ T cell antigens warrants further investigation.

In this study, we investigated whether ARFPs from the Gag, envelope (Env), and polymerase (Pol) regions elicit CD4+ T cell responses in individuals with chronic HIV-1 infection. We used an optimized *in vitro* expansion assay to assess T cell reactivity to ARFP-derived peptides, followed by intracellular cytokine staining to quantify antigen-specific cytokine production. Additionally, we performed epitope mapping to identify specific ARFP peptides recognized by CD4+ T cells and assessed their predicted binding to class II HLA alleles. Finally, we examined whether ARFP stimulation contributes to HIV-1 latency reversal.

Our findings provide new insights into the immunogenicity of HIV-1 ARFPs and their potential role in shaping CD4+ T cell responses. Understanding the contribution of ARFP-specific CD4+ T cells to the overall immune response against HIV-1 could inform vaccine design and therapeutic approaches aimed at targeting cryptic viral antigens.

## Methods

### Study participants

This study was approved by the Institutional Review Board (IRB) of Johns Hopkins University. Written informed consent was obtained from all participants prior to their enrollment. The study cohort included 13 people with HIV who were started on suppressive antiretroviral therapy during the chronic phase of infection (chronic progressors or CPs) and 6 HIV negative donors (HNDs). Peripheral blood mononuclear cells (PBMCs) were collected from whole blood after Ficoll-Paque PLUS gradient centrifugation. The median CD4 count of CPs was 830, with a range from 225 to 1550. The median age of participants was 53 years, with a range from 28 to 66 years. The median time on ART was 16 years, with a range from 2 to 23 years. Detailed characteristics of the participants are provided in **Supplementary Table 1**.

### Selection of alternative reading frame peptides

A total of 14 Gag ARFP, 7 Pol ARFP, and 3 Env ARFP peptides were included in this study. ARFP sequences were obtained from two sources. ARFP peptides were either selected from previously reported polypeptides (2, 3) or were derived from long uninterrupted polypeptide sequences within the Gag +1 and Gag +2 reading frames (**Table 1**). The peptides were synthesized by Genscript Biotech Corporation (Piscataway NJ) with a purity of > 85%.

**Table 1:** Alternate reading frame peptides used in this study.

Protein	Reading Frame	Polypeptide sequence	Peptide name	Sequence
Gag	+2	MGKSSRREGFQPRSDTHVFSIIRRSHPTRFKHHAKHSGGTSSSHANVKRRDHQ	Gag-ARFP 1	MGKSSRREGFQPRSDTH
			Gag-ARFP 2	SDTHVFSIIRRSHPTRF
			Gag-ARFP 3	PTRFKHHAKHSGGTSSS
			Gag-ARFP 4	GGTSSSHANVKRDHQ
Gag	+1	GRSGLPTREGQGIFFRADQSQQPHQKRASGLGKRQQLPLRSRSR	Gag-ARFP 5	GRSGLPTREGQIFFRAD
			Gag-ARFP 6	IFFRADQSQQPHQKRAS
			Gag-ARFP 7	HQKRASGLGKRQQLPLR
			Gag-ARFP 8	RASGLGKRQQLPLRSRS
Gag	+2	FFRENLAFPQGKAREFSSEQTRANSPTRRELQVWGRDNNSLSEAGADRQGTVSFSFPQITLWQRPLVTI	Gag-ARFP 9	FFREDLAFPQGKAREFS
			Gag-ARFP 10	KAREFSSEQTRANSPTR
			Gag-ARFP 11	ANSPTRRELQVWGRDNN
			Gag-ARFP 12	WGRDNNSLSEAGADRL
			Gag-ARFP 13	NNSLSEAGADRLRQGTV
		HRKQQPGQPKLPYSAE	Gag-ARFP 14	HRKQQPGQPKLPYSAE
Pol	+1	MAAISPVLRLRPPVGGRESSRNLEFPTIPKVKE	Pol-ARFP 1	MAAISPVLRLRPPVG
			Pol-ARFP 2	PPVGGRESSRNLEFP
			Pol-ARFP 3	SSRNLEFPTIPKVKE
		RHQGLDISTMCFHRDGKDHQQYSKVA	Pol-ARFP 4	TGTAEIHFGKDQQSFSGKVKGQ
			Pol-ARFP 5	RHQGLDISTMCFHRDGK
			Pol-ARFP 6	MCFHRDGKDHQQYSKVA
Pol	+2	TMAIDRRKNKSISRNLY	Pol-ARFP 7	TMAIDRRKNKSISRNLY
Env	+1	MGYLCGRKQPPLYFVHQMIKHMTQRYTMFGPHMPVYPQTPTHKK	Env-ARFP 1	MGYLCGRKQPPLYFVHQ
			Env-ARFP 2	FVHQMLKHMIQRYIMGF
			Env-ARFP 3	IMFGPHMPVYPQTPTHKK

### T cell expansion culture assays and polyfunctionality analysis

CD8-depleted PBMCs were cultured for 10 days in RPMI media with 10% Fetal Calf Serum (R10 media) supplemented with 10 IU/mL IL-2. The antiretroviral drug raltegravir was added at a concentration of 4uM to prevent viral replication. The cells were stimulated with 10 μg/mL of peptide pools (Gag ARFP, Env ARFP, and Pol ARFP) and DMSO. The initial cell count per condition ranged from 12 to 45 million PBMCs. Media replenishment was performed on days 3 and 7 by replacing half of the culture medium with fresh R10 media containing 10 IU/mL IL-2. After 10 days, cells were washed, replated in R10 media with 10 U/mL IL-2, and rested for at least 6 hours at 37°C before stimulation. For restimulation, cells were incubated with the same peptide pools (10 μg/mL) in the presence of protein transport inhibitors (GolgiPlug, BD Biosciences, 1 μg/mL; GolgiStop, BD Biosciences, 0.7 μg/mL) and co-stimulatory antibodies targeting CD28 (BD Biosciences) and CD49d (BD Biosciences). After a 16-hour incubation at 37°C, cells were washed and stained for surface markers using antibodies against CD3 (Pacific Blue, BioLegend), CD4 (PerCP-Cy5.5, BioLegend), and CD8 (BV-605, BioLegend). Following surface staining, cells were fixed, permeabilized, and stained intracellularly with antibodies targeting TNF-α (PE-Cy7, BD Biosciences), IFN-γ (APC, BD Biosciences), and IL-2 (PE, BioLegend). Flow cytometry was performed using a BD FACS LSR Fortessa flow cytometer, with at least 100,000 events collected within the lymphocyte gate. Data were analyzed in FlowJo software (version 10.10.0) to quantify cytokine-producing antigen-specific T cells.

In order to identify epitopes, an expansion assay was performed using Gag ARFP or Env ARFP peptide pools, where cells were cultured for 10 days before undergoing a 16-hour restimulation with individual Gag ARFP or Env ARFP peptides. This assay followed the same culture conditions and methodology described above.

Polyfunctionality of antigen-specific T cells was analyzed using FlowJo for intracellular cytokine staining and Boolean gating. Data were processed and exported for further visualization. Statistical analyses were performed using GraphPad Prism (version 10.4.1).

### HLA haplotyping and epitope binding predictions

High-resolution HLA class II typing was carried out by the Johns Hopkins Hospital Immunogenetics Laboratory. To assess peptide-MHC binding, in silico predictions were performed using the Immune Epitope Database (IEDB; http://www.iedb.org) between September 11 and 12, 2024. The IEDB-recommended 2.22 prediction algorithm was used, which applies a consensus approach that integrates NN-align, SMM-align, CombLib, and Sturniolo methods when available for the specific allele, or defaults to NetMHCIIpan when not (10, 11). Binding predictions were generated for the most immunogenic peptides. MHC class II alleles with a predicted percentile rank below 30 were included and are presented in **Table 2** in order of increasing percentile rank.

**Table 2:** MHC-II binding predictions for immunogenic ARFP peptides.

Participant	Peptide name	Peptide Sequence	Predicted MHC-II allele (IEDB analysis)
CP9	Gag ARFP 11	ANSPTRRELQVWGRDNN	DRB1*11:02; DRB4*01:03
CP86	Gag ARFP 9	FFREDLAFPQGKAREFS	DRB3*02:02; DRB5*01:01
	Gag ARFP 10	KAREFSSEQTRANSPTR	DRB3*02:02; DRB5*01:01; DPA1*01:03/DPB1*01:01; DPA1*02:01/DPB1*02:01
CP97	Gag ARFP 1	MGKSSRREGFQPRSDTH	DPA1*01:03/DPB1*02:01
	Env ARFP 2	FVHQMLKHMIQRYIMGF	DRB1*13:02
CP101	Gag ARFP 1	MGKSSRREGFQPRSDTH	DPA1*01:02/DPB1*05:01; DPA1*01:03/DPB1*02:01
	Env ARFP 2	FVHQMLKHMIQRYIMGF	DRB1*12:01; DRB1*13:02

### ELISpot assay

An interferon-gamma (IFN-γ) ELISpot assay was performed using individual peptides spanning Gag ARFP and Env ARFP. There were 14 individual Gag ARFP peptides and 3 individual Env ARFP peptides. Each peptide was used at a final concentration of 10 μg/mL and incubated with 200,000 CD8-depleted PBMCs/well in RPMI media supplemented with 10% fetal bovine serum for 20 hours at 37°C, following the manufacturer’s instructions. The plates were incubated for 20 hours with the individual Gag ARFP and Env ARFP peptides. Two replicates were performed for each peptide.

ELISpot plates were analyzed by an independent investigator using the AID iSpot Spectrum and vendor-supplied software. The software quantified IFN-γ spot-forming units (SFUs) per well. SFU/million cells was calculated by adjusting the number of spots per well with the appropriate dilution factor. A positive response was defined as a mean SFU of ≥30.

### HIV RNA quantification

Supernatant samples were collected on day 7 after stimulation with Gag ARFP, Env ARFP, or Pol ARFP peptide Pools. RNA was extracted using the Zymo Quick-RNA Viral Kit according to the manufacturer’s instructions. Briefly, 200 μL of supernatant was mixed with DNA/RNA Shield and processed through Zymo-Spin IC Columns with successive washes using Viral Wash Buffer and ethanol. RNA was eluted in DNase/RNase-Free water and stored at -80°C until further processing.

Complementary DNA (cDNA) was synthesized using qScript cDNA SuperMix (Quanta Biosciences). Reverse transcription was performed under standard cycling conditions (25°C for 5 min, 42°C for 30 min, 85°C for 5 min, and 4°C hold). The resulting cDNA was used for HIV RNA quantification as previously described (12) to assess latency reversal following ARFP peptide stimulation. The limit of detection for HIV RNA was 300 copies/ml.

## Results

### CD4+ T cells from CPs recognize ARFP peptide pools

**Figure 1:**
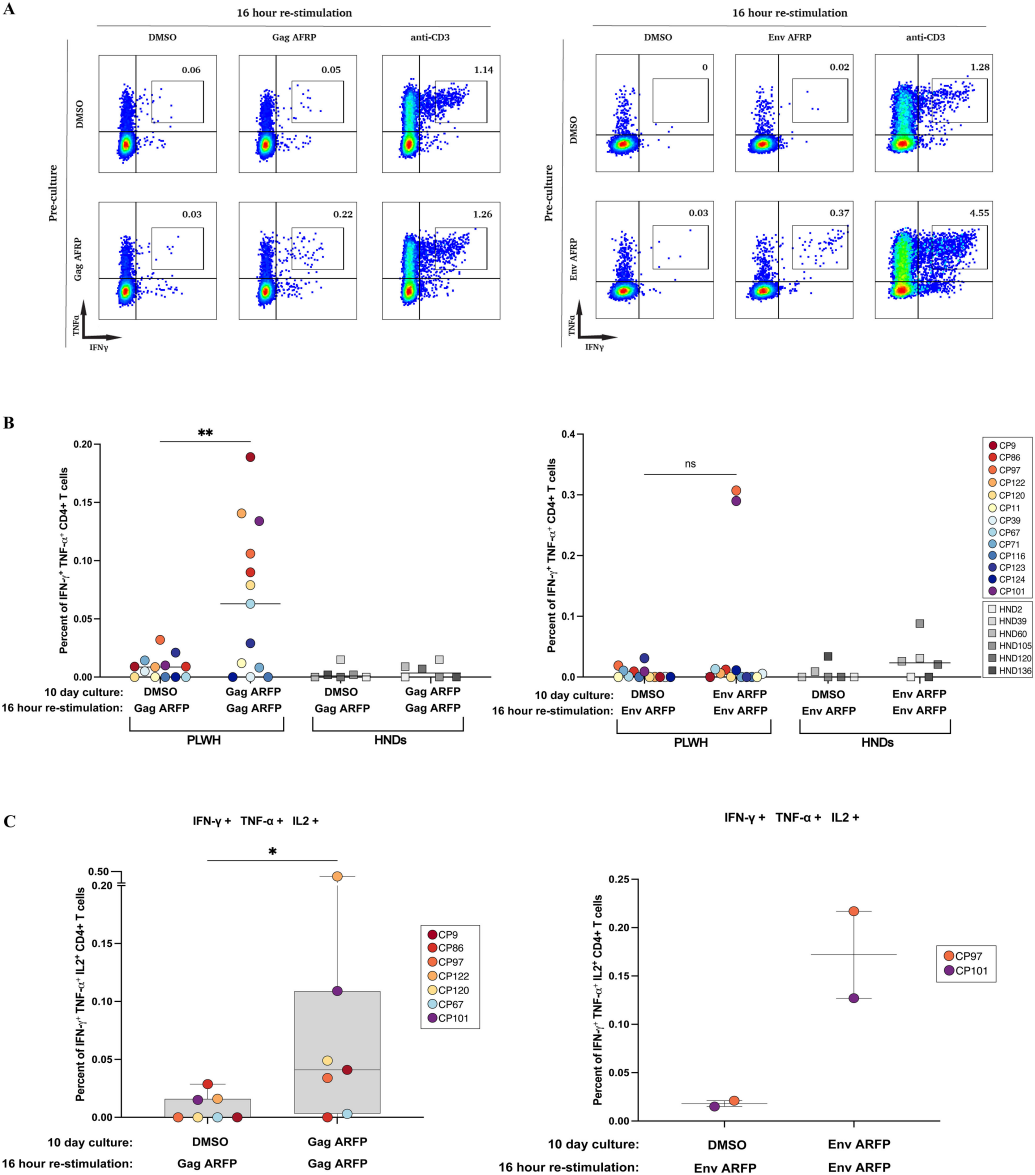
Functional characterization of antigen-specific CD4+ T cell responses following in vitro expansion. **(A)** PBMCs from two participants (CP9 and CP97) were pre-cultured for 10 days with either DMSO (top row) or Gag ARFP (left bottom row) or Env ARFP peptides (right bottom row). After expansion, cells were stimulated for 16 hours with DMSO, Gag ARFP peptides, Env ARFP peptides or anti- CD3 antibodies and stained for IFN-γ and TNF-α expression. The percentages in each quadrant indicate the proportion of cells expressing the respective cytokines. **(B)** The percentage of IFN-γ+ TNF-α+CD4+ T cells was measured in 19 participants (13 CPs & 6 HNDs) after a 10-day pre-culture and 16-hour restimulation with Gag ARFP or Env ARFP peptide pools, with DMSO as the negative control. **p<0.01, ns, not significant. **(C)** The polyfunctional capacity of CD4+ T cells was assessed by measuring the co-expression of IFN-γ, TNF-α, and IL-2 in response to Gag ARFP and Env ARFP peptides. The figure shows the proportion of cells expressing these cytokines, highlighting the polyfunctional nature of the immune response in responders. *p<0.05 .

HIV-1 alternate reading frame proteins (ARFPs) have been shown to elicit CD8+ T cell responses. However, it is not known whether they are also recognized by CD4+ T cells. To investigate this, CD8+ T cell-depleted PBMCs from 13 CPs and 6 HNDs were stimulated with Gag, Pol or Env ARFP ARFP peptide pools for 10 days, followed by a 16-hour restimulation with the same peptide pools. Cytokine production was assessed using intracellular cytokine staining and flow cytometry. **Figure 1A** illustrates the ARFP Gag peptide pool response in CP9, where PBMCs were cultured for 10 days with either ARFP peptides or DMSO. Restimulation with Gag ARFP peptides resulted in a marked increase in IFN-γ+ TNF-α+ CD4+ T cells (0.22%) compared to DMSO-cultured cells (0.05%). A similar response was observed in CP97, where restimulation with the Env ARFP peptide pool led to an increase in IFN-γ+ TNF-α+ CD4+ T cells (0.37%) compared to DMSO-cultured cells (0.02%).

Following restimulation with Gag ARFP peptides, significant increases in IFN-γ+ TNF-α+ CD4+ T cells were observed in 7 out of 13 CPs (median 0.06%, range 0.0%–0.19%) compared to cells cultured with DMSO for 10 days (median 0.01%, range 0%–0.02%). Env ARFP peptides elicited IFN-γ+ TNF-α+ CD4+ T cell responses in 2 of 13 CPs, with response frequencies of 0.29% and 0.31%, compared to 0.01% and 0.02% in cells cultured with DMSO for 10 days. In contrast, neither Gag nor Env ARFP peptides were recognized by CD4+ T cells from any of the 6 HNDs (**Figure 1B**). Additionally, none of the 13 CPs exhibited significant IFN-γ+ TNF-α+ CD4+ T cell responses upon restimulation with the Pol ARFP peptide pool (**Supplementary Figure 1**).

To further investigate the polyfunctional capacity of CD4+ T cells, we assessed the co-expression of IFN-γ, TNF-α, and IL-2 following restimulation with Gag and Env ARFP peptide pools. A significant increase in T cells recognizing all three cytokines was observed in the 7 Gag ARFP responders and 2 Env ARFP responders. In the Gag ARFP responders, the median percentage of IFN-γ+ TNF-α+ IL-2+ CD4+ T cells was 0.04% following 10-day culture with Gag ARFP, compared to 0% for cells cultured with DMSO. Similarly, the two Env ARFP responders exhibited increases in polyfunctional responses, with 0.22% and 0.13% of IFN-γ+ TNF-α+ IL-2+ CD4+ T cells following 10-day culture with Env ARFP, compared to 0.02% with DMSO (**Figure 1C**).

### Identification of ARFP epitopes

**Figure 2:**
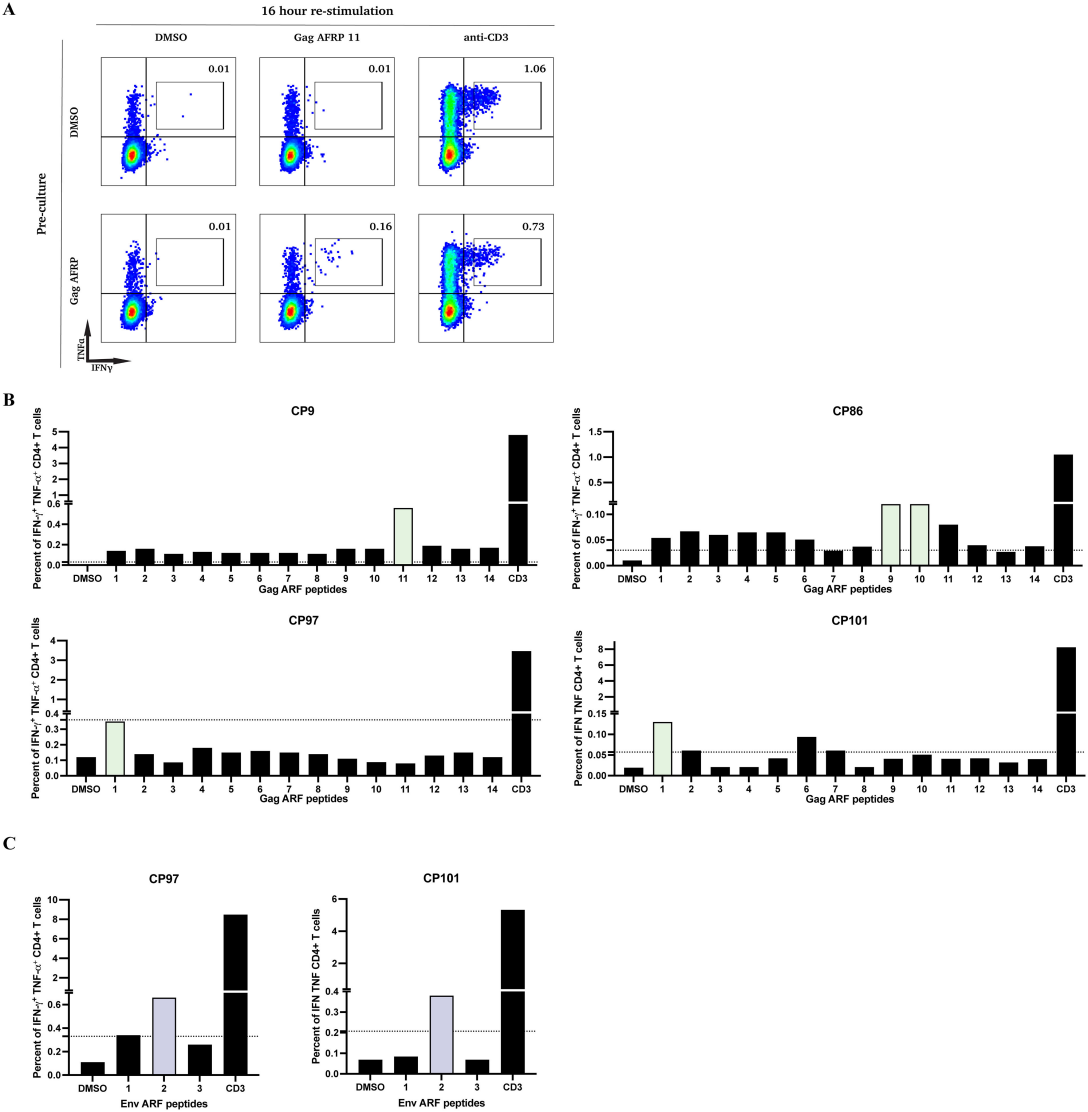
Functional characterization of CD4+ T cell responses to individual ARFP peptides following in vitro expansion. **(A)** PBMCs from one participant (CP9) were pre-cultured for 10 days with either DMSO (top row) or Gag ARFP (bottom row). After expansion, cells were stimulated for 16 hours with DMSO, Gag ARFP 11 peptides, or anti-CD3 antibodies and stained for IFN-γ and TNF-α expression. The percentages in each quadrant indicate the proportion of cells expressing the respective cytokines. **(B)** PBMCs from 4 participants were pre-cultured for 10 days with either Gag ARFP or Env ARFP. After expansion, cells were stimulated for 16 hours with DMSO, individual Gag ARFP peptides, individual Env ARFP peptides **(C)**, or anti-CD3 antibodies and stained for IFN-γ and TNF-α expression. The percentage of IFN-γ+ TNF-α+CD4+ T cells was measured in the 4 participants. The colored bars represent the most immunogenic peptides. The dotted line represent the threshold for a positive response (SI >3).

The next objective was to define the specific ARFP peptides targeted by CD4+ T cells. To assess this, we cultured CD8+ T cell-depleted PBMCs from 4 CPs that responded to Gag ARFP peptide pool stimulation and 2 CPs that responded to Env ARFP peptide pool stimulation over 10 days. We then restimulated these cells with individual peptides from Gag ARFP and Env ARFP for 16 hours, and then we measured cytokine production by intracellular cytokine staining and flow cytometry analysis. **Figure 2A** illustrates this response in CP9, where PBMCs cultured with Gag ARFP peptides for 10 days exhibited a marked increase in IFN-γ+ TNF-α+ CD4+ T cells (0.16%) upon restimulation with Gag ARFP peptide 11, compared to DMSO-cultured cells (0.01%).

Across CPs that responded to Gag ARFP or Env ARFP peptide pools, a few individual peptides elicited IFN-γ and TNF-α production, with variability in the number of recognized peptides across participants ranging from 1–2 individual peptides (**Figures 2B, C**).

### Effector T cell responses to ARFP peptides are absent in circulation

To determine whether ARFP-specific T cells exist within the circulating effector T cell pool, an ELISpot assay was performed in three CPs using 14 individual Gag ARFP peptides and 3 individual Env ARFP peptides. In contrast to the cytokine-producing memory T cell responses observed in expanded cultures, no effector IFN-γ responses were detected across all three CPs in response to the individual peptides suggesting that ARFP-specific CD4+ T cells were present at a low frequency (**Supplementary Figure 2**).

### Immunogenic ARFP peptides are predicted to bind to CP MHC II alleles

Given the identification of immunogenic ARFP peptides, we next investigated whether these peptides are predicted to bind MHC-II alleles in CPs. High-resolution class II HLA typing was performed in five CPs who exhibited responses to either Gag ARFP or Env ARFP peptides. Using an in silico binding prediction algorithm, we identified multiple class II alleles with high predicted binding affinity for the most immunogenic ARFP peptides. Each CP exhibited unique MHC-II alleles capable of binding at least one ARFP peptide (**Table 2**).

### Reversal of latency by ARFPs in some CPs

**Figure 3:**
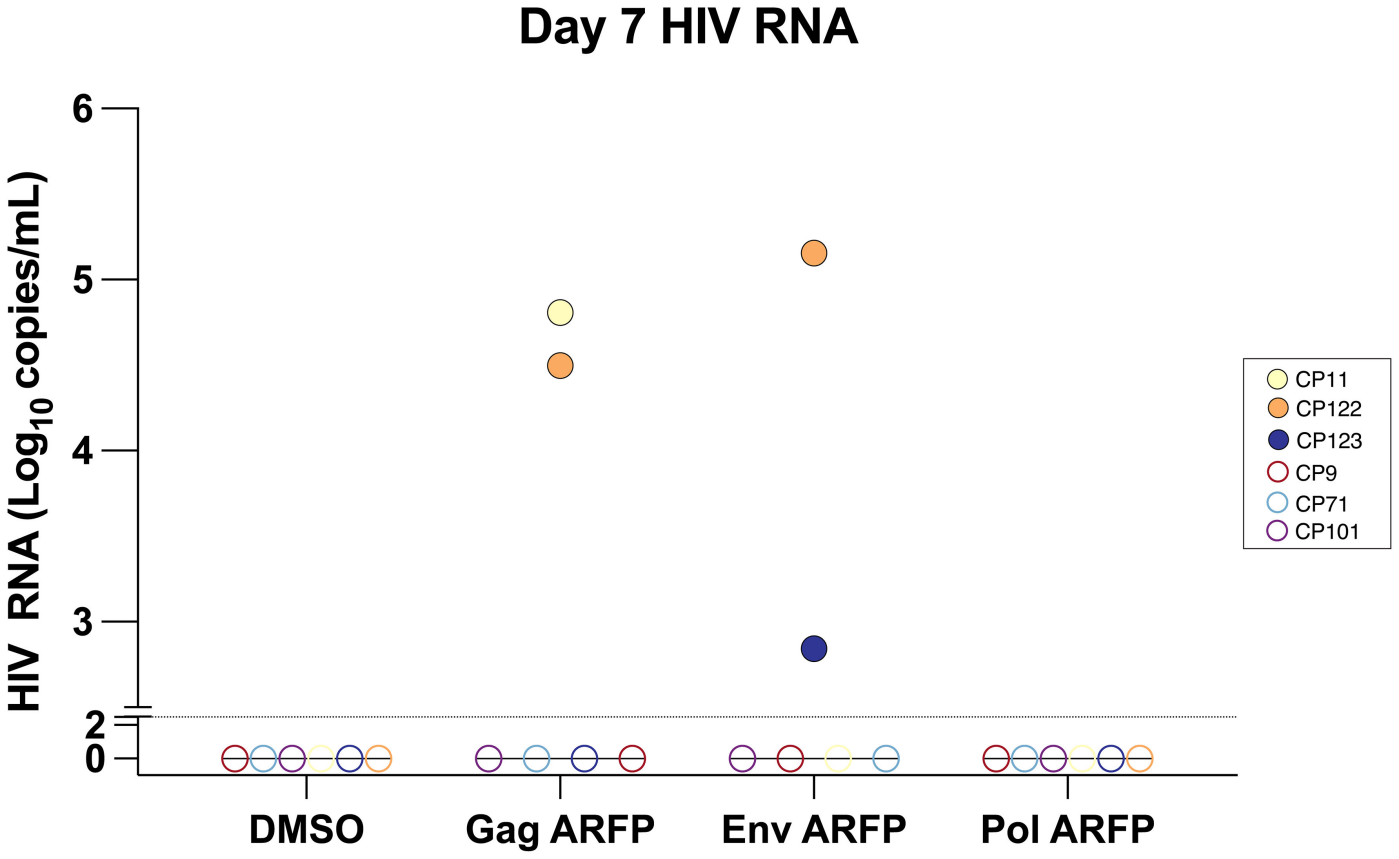
Quantification of HIV RNA levels in culture supernatant after AFRP peptide stimulation. PBMCs from 6 participants were cultured with either Gag ARFP or Env ARFP or Pol ARFP. On day 7, the HIV RNA levels were measured in 6 CPs. The dotted horizontal line represents the limit of detection of 300 copies/ml for HIV RNA. Open symbols represent values that were below the limit of detection.

To evaluate whether ARFP peptides contribute to HIV latency reversal, viral RNA was quantified from culture supernatants collected on day 7 from 6 CPs following stimulation with Gag, Env, or Pol ARFP peptide pools. Latency reversal, indicated by an increase in HIV RNA, was observed in response to Gag ARFP peptides in 2 of 6 participants, with viral RNA levels reaching 31,613.7 copies/mL and 64,484.5 copies/mL. Similarly, Env ARFP peptides induced latency reversal in 2 of 6 participants, with RNA levels of 698.1 copies/mL and 143,464 copies/mL. In contrast, stimulation with Pol ARFP peptides failed to induce latency reversal in any of the 6 CPs (**Figure 3**). The lack of correlation between the magnitude of ARFP-specific T cell responses and latency reversal could be explained by the fact that not all ARFP-specific T cells are latently infected.

## Discussion

In this study, we demonstrate that HIV-1 alternative reading frame proteins (ARFPs) elicit CD4+ T cell responses in people living with HIV (PLWH), expanding on prior findings that have largely focused on ARFP-specific CD8+ T cell responses. By stimulating CD8-depleted PBMCs with ARFP-derived peptide pools, we identified IFN-γ+ TNF-α+ CD4+ T cell responses in a subset of CPs.

ARFP-derived peptides arise through alternative translation mechanisms, including ribosomal frameshifting, leaky scanning, and upstream ORFs, all of which have been shown to generate cryptic epitopes in viral infections (7, 13). While most studies of HIV antigen presentation focus on canonical protein sequences, recent work has demonstrated that HIV-infected cells can present noncanonical peptides via MHC-I, contributing to CD8+ T cell recognition (1–3). However, the role of CD4+ T cells in ARFP-specific immunity has remained unexplored. Our findings demonstrate that CD4+ T cells can recognize ARFP peptides, broadening the known HIV immunopeptidome. A recent study (14) used ribosomal profiling (Riboseq) and mass spectrometry-based immunopeptidomics to identify 98 ARFs encoding sORFs in HIV-infected CD4+ T cells. These ARFP-derived peptides were detected throughout the HIV genome, including UTR regions, and were highly conserved among HIV-1 clade B and C strains. Some ARF-encoded sequences were even more conserved than canonical coding sequences, and ARFP-derived peptides were shown to elicit polyfunctional T cell responses in both CD4+ and CD8+ T cells. In our study, expanded ARFP-specific CD4+ T cells also displayed polyfunctionality, with a significant proportion of cells producing multiple cytokines (IFN-γ, TNF-α, and IL-2), indicative of an effective immune response (15–17). These findings support the hypothesis that ARFPs may play a significant role in HIV immune responses.

The ability of ARFP-derived peptides to elicit CD4+ T cell responses has important implications for HIV vaccine design and therapeutic immune interventions. Given that robust HIV-specific CD4+ T cell responses have been linked to better viral control and slower disease progression (18), targeting cryptic epitopes derived from ARFPs could offer a new avenue for vaccine development. Prior studies have shown that a substantial frequency of latently infected cells recognize viral antigens including HIV Gag (19–21). The ability of some ARFP peptides to induce HIV RNA expression in culture suggest that some latently infected cells also recognize ARFPs and this may have implications for latency-reversal strategies. Further studies are needed to determine whether ARFP-derived peptides are naturally processed and presented *in vivo* and whether they contribute to HIV reservoir maintenance or immune surveillance.

While this study provides evidence of CD4+ T cell responses to ARFP-derived peptides, there are several limitations that warrant further investigation. The relatively small sample size limits the generalizability of our findings. Another limitation is the use of *in vitro* peptide stimulation, which does not fully recapitulate natural antigen processing and presentation. However, the presence of ARFP-specific memory responses in CPs does suggest that T cells have encountered these antigens *in vivo*. Furthermore, our HLA typing and binding predictions support the likelihood that the identified peptides are presented via MHC-II, providing additional evidence of their potential relevance *in vivo*. Patients on ART experience a significant decline in circulating HIV-specific effector T cells (22) thus an expansion assay is needed to detect low frequency memory responses. However, the *in vitro* culture of T cells may significantly alter their phenotype and function. A direct comparison between responses to ARFs and canonical reading frames would help clarify the biological relevance of each response. It would also be ideal to analyze these responses in viremic individuals given the higher frequency of HIV-specific cells seen in these individuals. Follow-up studies should investigate whether ARFP-specific CD4+ T cell responses correlate with clinical outcomes in PLWH and whether targeting these cryptic epitopes could enhance immune-mediated viral control.

This study establishes that HIV-1 ARFPs elicit CD4+ T cell responses, advancing our understanding of noncanonical HIV antigens. Our findings underscore the importance of further research into alternative translation products, their relevance for HIV vaccine design, and their potential role in latency reversal and immune surveillance.

## Data Availability

The original contributions presented in the study are included in the article/**Supplementary Material**. Further inquiries can be directed to the corresponding author.
