# Supplementary material for: CD4+ T cell recognition of HIV-1 alternate reading frame proteins
Source: Front Immunol. 2025 May 22;16:1600132. doi: 10.3389/fimmu.2025.1600132 (PMC12137346; doi:10.3389/fimmu.2025.1600132)
Supplement: Supplementary file 1 [file DataSheet1.pdf]

Supplementary Table 1: Clinical characteristics of study participants

Study participants	Age, gender	CD4 count (cells/ul)	Viral load (copies RNA/ml)	Years on suppressive antiretroviral regimen	Antiretroviral regimen
CP9	66, M	935	< 20	23	Emtricitabine-Tenofovir alafenamide Bictegravir
CP11	60, M	1550	<20	17	Emtricitabine-Tenofovir alafenamide Bictegravir
CP39	63, M	915	< 20	20	Emtricitabine-Tenofovir alafenamide Dolutegravir
CP67	65, M	471	<20	18	Emtricitabine-tenofovir Alafenamide Bictegravir
CP71	64, M	370	<20	16	Dolutegravir Rilpivirine
CP86	55, M	225	<20	2	Rilpivirine Cabotegravir
CP97	53, F	1003	<20	17	Rilpivirine Cabotegravir
CP101	38, M	830	<20	12	Emtricitabine-Tenofovir alafenamide Darunavir/ritonavir
CP116	28, M	848	<20	3	Rilpivirine Cabotegravir
CP120	34, M	561	<20	17	Rilpivirine Cabotegravir
CP122	43, M	832	20	5	Emtricitabine-tenofovir Alafenamide Bictegravir
CP123	36, F	670	<20	3	Rilpivirine Cabotegravir
CP124	51, M	708	<20	7	Rilpivirine Cabotegravir

Supplementary figure 1

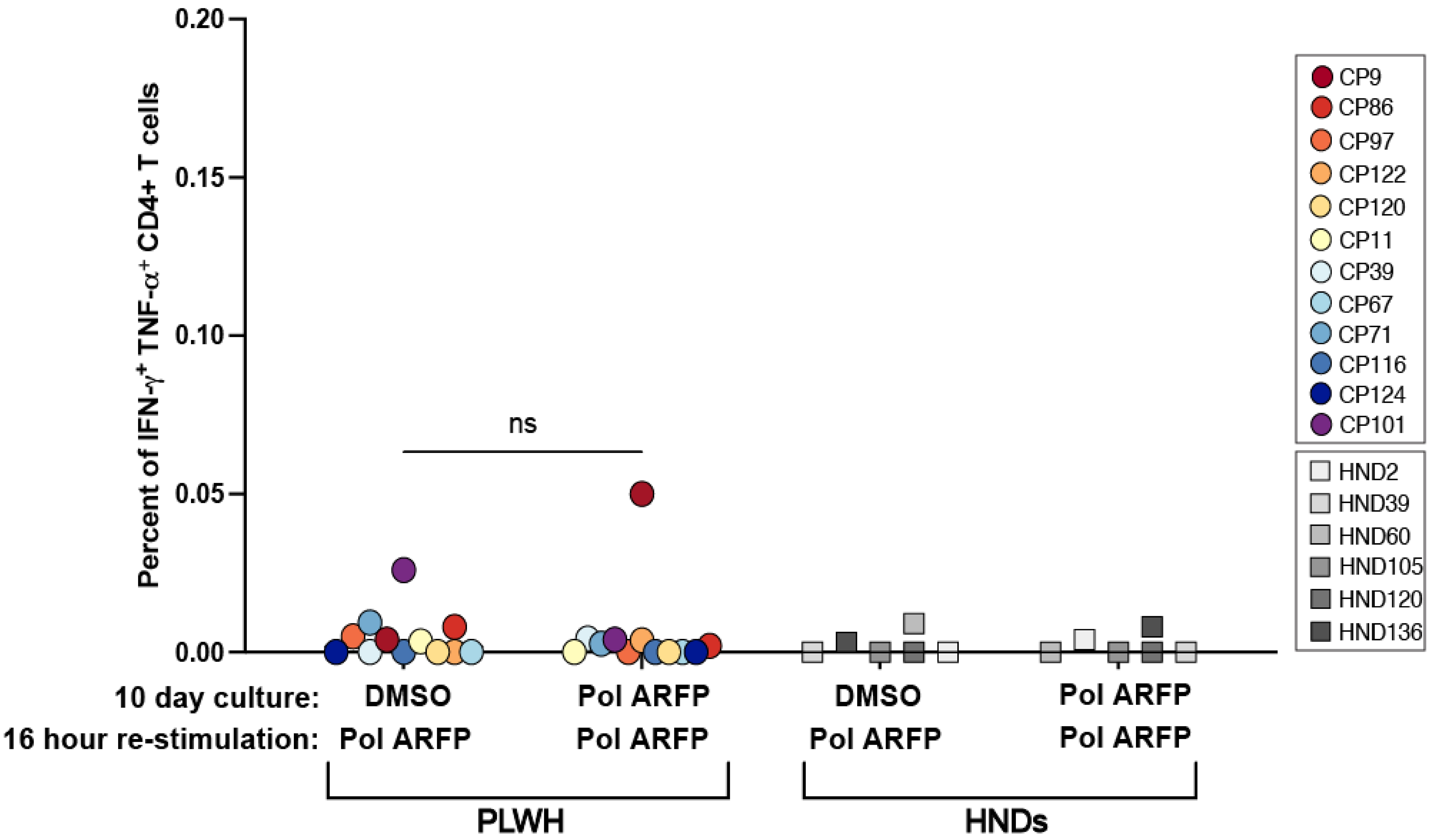

Supplementary figure 1: Functional characterization of Pol ARFP-specific CD4+ T cell responses following in vitro expansion.

The percentage of IFN-γ+ TNF-α+ CD4+ T cells was measured in 18 participants (12 CPs & 6 HNDs) after a 10-day pre-culture and 16-hour restimulation with Pol ARFP peptide pool, with DMSO as the negative control. ns, not significant

Supplementary Figure 2

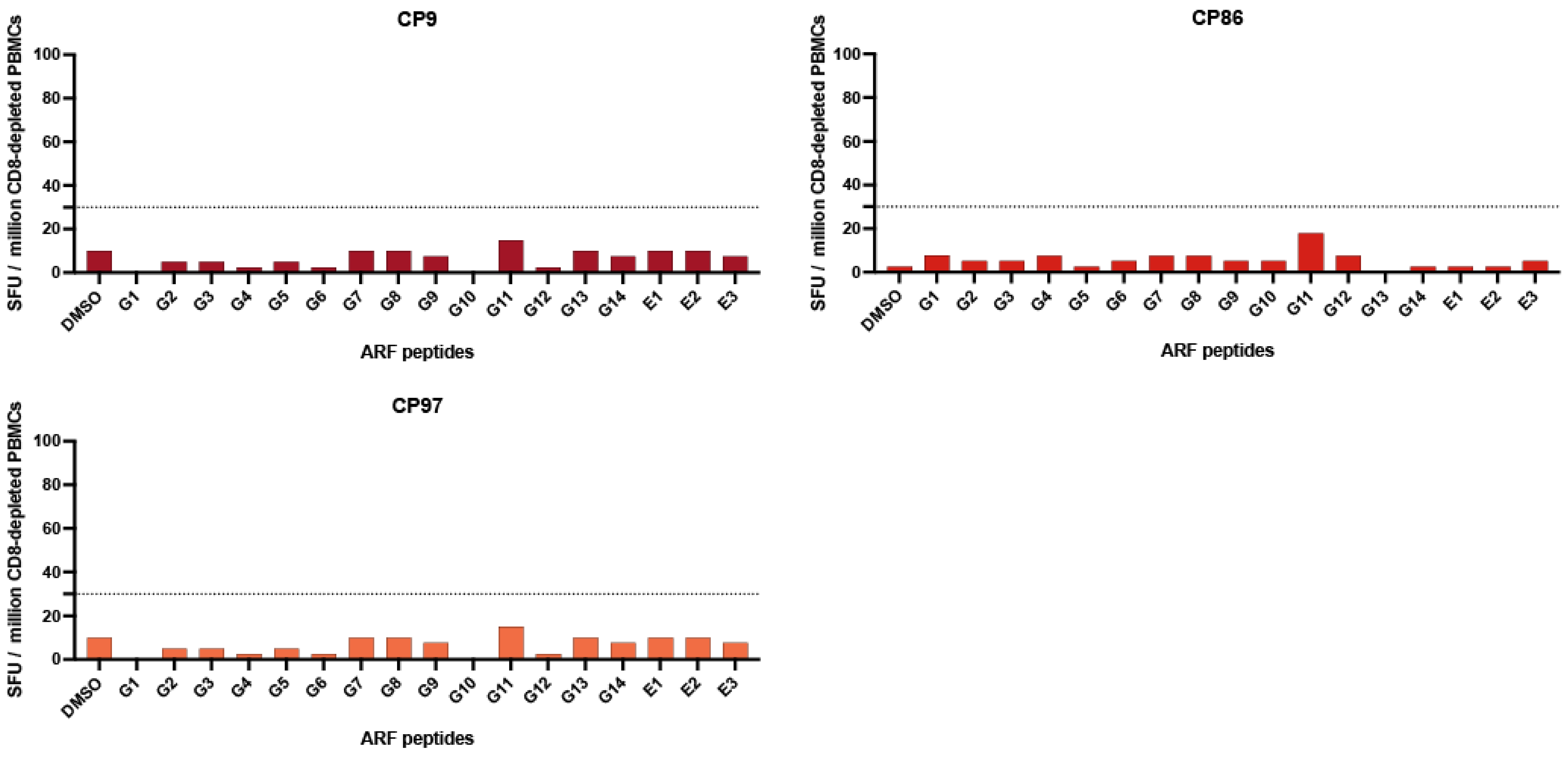

Supplementary Figure 2: Quantification of circulating effector CD4+ T cell responses to individual ARFP peptides using IFN-γ ELISpot.
PBMCs from 3 CPs were stimulated with 14 individual Gag ARFP peptides and 3 individual Env ARFP peptides in an IFN-γ ELISpot assay. The number of spot-forming units (SFU) per million PBMCs was quantified for each peptide. The dotted horizontal line represents the threshold for a positive response (SFU ≥ 30).
